# Recent Advances in Multifunctional Hydrogels for the Treatment of Osteomyelitis

**DOI:** 10.3389/fbioe.2022.865250

**Published:** 2022-04-25

**Authors:** Weiwei Xin, Yingjian Gao, Bing Yue

**Affiliations:** Department of Orthopedics, Renji Hospital, School of Medicine, Shanghai Jiao Tong University, Shanghai, China

**Keywords:** hydrogel, osteomyelitis, infection, drug-loaded materials, *in vivo*

## Abstract

Osteomyelitis (OM), a devastating disease caused by microbial infection of bones, remains a major challenge for orthopedic surgeons. Conventional approaches for prevention and treatment of OM are unsatisfactory. Various alternative strategies have been proposed, among which, hydrogel-based strategies have demonstrated potential due to their unique properties, including loadable, implantable, injectable, printable, degradable, and responsive to stimuli. Several protocols, including different hydrogel designs, selection of antimicrobial agent, co-administration of bone morphogenetic protein 2 (BMP 2), and nanoparticles, have been shown to improve the biological properties, including antimicrobial effects, osteo-induction, and controlled drug delivery. In this review, we describe the current and future directions for designing hydrogels and their applications to improve the biological response to OM *in vivo*.

## 1 Introduction

### 1.1 Osteomyelitis

Osteomyelitis (OM) is a catastrophic disease caused by a pathogen infection. The incidence of OM varies from 0.1 to 30% in different subspecialties, costing the health care system between $15,000–$17,000 per patient (Schwarz et al., 2019). Staphylococcal infections account for 75% of cases (Arciola et al., 2005; Walter et al., 2012). The most common pathogen is *Staphylococcus aureus* (*S. aureus*) (Darouiche, 2004; Arciola et al., 2005; Pulido et al., 2008), and over 50% of the infections are caused by methicillin-resistant *Staphylococcus aureus* (MRSA) (Kaplan, 2014). The invasive capability of *S. aureus* is attributed to its virulence factors and resistance mechanisms, including toxin secretion (Otto, 2014), adherence (Otto, 2008), the slow-growing small colony variant (SCV) subpopulation (Sendi et al., 2006; Tuchscherr et al., 2010), intracellular persistence in bone cells (Masters et al., 2019), the development of antimicrobial resistance (Kaplan, 2014), and biofilm formation (de Mesy Bentley et al., 2017; de Mesy Bentley et al., 2018; Ricciardi et al., 2018; Masters et al., 2019). These mechanisms enable bacteria to endure in hostile environments (Mah and O'Toole, 2001; Savage et al., 2013; de Mesy Bentley et al., 2017; de Mesy Bentley et al., 2018) and cause damage to the surrounding host tissue *via* various mechanism (Redlich and Smolen, 2012; Junka et al., 2017; Putnam et al., 2019).

Currently, no ideal classification and treatment strategies for OM are available. Due to the refractory nature of OM, its costly and long-term treatment, and high rate of disability, improved treatment strategies are urgently needed. Although some therapeutic strategies have been investigated, such as vaccines and monoclonal antibodies (mAbs) (Fowler and Proctor, 2014; Rouha et al., 2015; Thammavongsa et al., 2015; Varshney et al., 2018), the identification of bacterial species and sensitive antibiotics, removal of the implants, extensive debridement, and systemic antibiotic administration for 4–6 weeks remain the standard treatment strategies for OM. Irrigation with antiseptic and implantation of antibiotic-laden spacers in the dead space are adjuncts to this standard procedure (Peng et al., 2010).

Under all treatment scenarios, the key to success is the thorough debridement of infected and necrotic tissue, and eliminating patient risk factors (such as smoking, diabetes mellitus, and immunosuppressants). Unfortunately, it is not possible to completely debride the infectious tissues (Varshney et al., 2018). Clinically, bone debridement is performed by visually distinguishing necrotic “white” bone from healthy “red” bone. In this way, bacteria colonized in biofilms, especially in the osteocyte-lacuno canalicular network (OLCN) (de Mesy Bentley et al., 2017; de Mesy Bentley et al., 2018), remain in the “red” bone, thereby causing a recurrence of infection following multiple extensive debridement. The rate of failure for the staged revision approach against MRSA orthopedic device-related infections (ODRI) ranges between 11% and 52% (Parry and Duncan, 2014), which indicates the necessity for research on novel treatment strategies (Varshney et al., 2018).

Moreover, the effectiveness of the current treatments against *S. aureus* colonized in the bone matrix are currently unknown, although long-term use of systemic antibiotics is the current standard of care. Impaired local blood supply and multiple bacterial resistance mechanisms result in insufficient local concentrations of antibiotics following systemic administration. Meanwhile, a high dose of systemic antibiotic administration over an extended period is not recommended due to the potential systemic toxicity, including ototoxicity and nephrotoxicit (Naughton, 2008; Elyasi et al., 2012; Kubin et al., 2012).

To address these issues, topical antibiotic delivery, which can offer higher concentrations at the target site with fewer side effects compared to systemic administration (Hake et al., 2015), is a promising approach to manage the dead space due to the local drug infusion and its barrier effect on preventing biofilm formation (Pan et al., 2018).

### 1.2 Polymethylmethacrylate Antibiotic-Laden Bone Cement

As an antibiotic carrier, polymethylmethacrylate (PMMA) bone cement has been used to treat OM and ODRI. Implanted in the dead space, antibiotic-laden bone cement (ALBC) delivers a high concentration of antibiotics locally to the infection site without off-target systemic toxicity. However, as a local antibiotic vehicle, PMMA also has several drawbacks.

One limitation of PMMA is its poor drug-release kinetics. The molecular structure of PMMA and its tight packing influence the diffusion of drugs, and hence the release rate of the laden antibiotics. A large amount remains encapsulated within the polymer and is not released (Frutos Cabanillas et al., 2000; Kuehn et al., 2005). *In vitro* and *in vivo* findings have demonstrated that only 5%–18% of the incorporated drug is eluted from PMMA (Masters et al., 2019). Furthermore, the antibiotic release from PMMA presents a burst pattern within the first 24 h followed by a rapid decrease in release that lowers drug concentrations to below the minimum inhibitory concentration (MIC) (Guelcher et al., 2011). Clinically, cement spacers remain *in situ* for up to 6 weeks. The sustained elution of antibiotics at such low concentrations can induce the formation of antibiotic resistance and small colony variants (SCVs) (Sendi et al., 2006; Masters et al., 2019). Additionally, the non-eluting surface of spacers and beads provides a site for pathogen colonization and biofilm formation (Barth et al., 2011; Ma et al., 2018). Weber and Lautenbach (1986) showed that gentamicin ALBC increased the percentage of drug-resistant bacteria from 29% preoperatively to 41% postoperatively. Furthermore, the exothermic reaction inactivates heat-sensitive antibiotics during polymerization of bone cement (Boot et al., 2021). The limited antibiotics that can be loaded in PMMA are not fully effective against the microorganisms. Moreover, unreacted methacrylate monomers can induce toxicity issues (Yoshii, 1997). Finally, beads and spacers require additional surgeries for removal.

**FIGURE 1:**
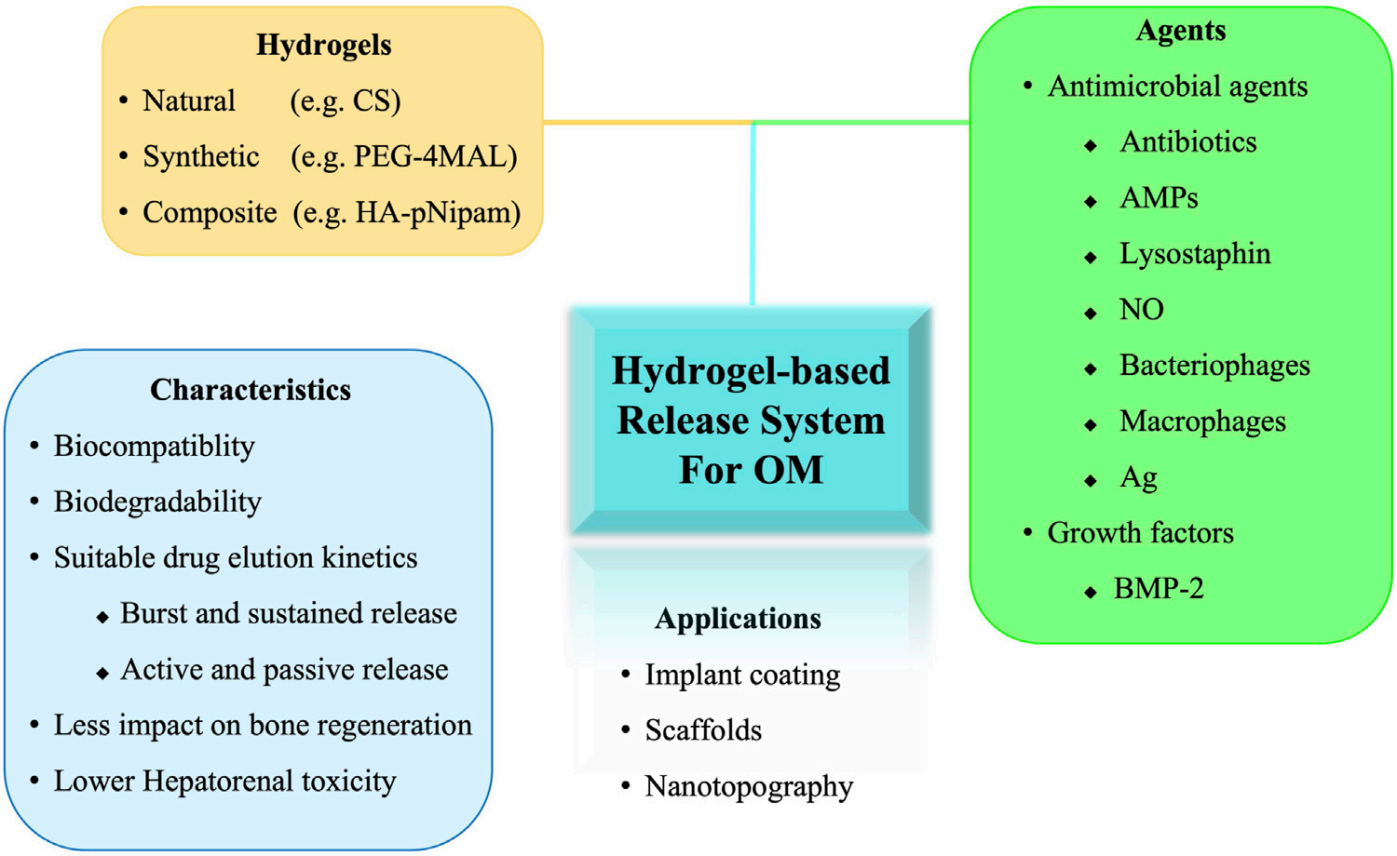
Schematic illustration of hydrogel-based release system for the treatment of osteomyelitis. OM, osteomyelitis; CS, Chitosan; PEG-4MAL, four-arm poly (ethylene glycol)-maleimide; HA-pNipam, Hyaluronic acid -poly (N-isopropylacrylamide); AMPs, Antimicrobial peptides; Ag, Silver; BMP, bone morphogenetic protein.

These drawbacks of PMMA bone cements have prompted the development of novel vehicles for topical drug delivery and dead space management, with the following criteria: 1) biocompatibility, 2) biodegradability, 3) suitable drug elution kinetics (i.e., burst release followed by sustained release at concentrations higher than the MIC), and 4) osteo-conductivity (Sarigol-Calamak and Hascicek, 2018). Various materials have potential for delivering antibiotics directly to complex microenvironments at desired concentrations, including calcium sulfates or phosphates, demineralized bone matrix, natural polymers, and synthetic polymers (Bibbo and Patel, 2006; Rupprecht et al., 2007; Borkhuu et al., 2008; Ferguson et al., 2014; Romano et al., 2014; Cho et al., 2018; Masters et al., 2019; Li et al., 2020b; Boot et al., 2020; Boot et al., 2021; Eltawila et al., 2021; Sun et al., 2021). Furthermore, studies have also investigated the delivery of alternative antimicrobial agents, combinations of antimicrobial agents, and osteoinductive adjuvants that are not effectively carried or eluted by PMMA (Inzana et al., 2016). Hydrogel-based release system have demonstrated potential due to their unique properties, such as high water absorption capacity, high porosity, almost free diffusible interconnectivity, and stimulus-responsive. Different agents or techniques have been evaluated in the complex microenvironment of OM. However, the *in vivo* data on hydrogels for the treatment of OM are limited. In this review, we describe the current and future directions for the design of hydrogels and their applications *in vivo* to improve the treatment of OM (Figure 1).

## 2 Hydrogels

Hydrogels are water-swollen polymer networks. For drug delivery, hydrogels combine the advantages of minimally invasive application, *in situ* polymerization, inherently adhesiveness, and sustained drug release (De Witte et al., 2018). Moreover, the protease-degradable nature of hydrogels, suitable for bone biological engineering, such as collagen, hyaluronic acid (HyA), also allows the host to degrade and replace them with repair tissue. Additional surgeries are not required to remove hydrogels, as is the case for non-degradable scaffolds (Johnson et al., 2018). These properties confer the significant therapeutic versatility of hydrogels for topical drugs delivery in heterogeneous and anatomically complex conditions, such as OM (Slaughter et al., 2009). Natural polymers, synthetic polymers, and the composite biomaterials have been used as drug vehicles in *in vivo* studies on OM (Supplementary Table S1).

### 2.1 Natural Polymers

Natural polymer-based hydrogels, such as collagen, HyA, and chitosan (CS), are both biocompatible and biodegradable, making them suitable for drug delivery (Liu et al., 2020).

#### 2.1.1 Collagen

Collagen is the most widely used natural polymer in orthopedic applications, among which Type I collagen is the most abundant structural protein in the body. As an important extracellular matrix protein of bone, Type I collagen can induce tissue regeneration. The early deposition of collagen matrix is essential for bone repair and remodeling, which forms a guiding structure for regeneration process. The interaction of collagen matrix with heterodimeric integrin receptors activates intracellular signal transduction pathways to induce various cellular proliferation, differentiation, and other functions. Thus, osteoblastic phenotypes are affected by collagen; cells migrate to collagen, where they organize and remodel the cytoskeleton (Rupprecht et al., 2007). For most local drug delivery systems using collagen, the collagen products are impregnated with an antibiotic solution, and the drugs are absorbed by the hydrophilic matrix (Inzana et al., 2016).

In a rabbit mandibular OM model with spontaneous oral bacterial contamination induced by arsenic trioxide, a single intra-lesion injection of gentamicin-collagen (GNT-COLL) hydrogels was more efficient at suppressing of OM than multidose systemic gentamicin. The OM was successfully halted, without recurrence for up to 12 weeks, where a significant increase in ridge length preservation percentage (RLP%) in the GNT-COLL hydrogel group was observed. These results showed up clinically as a slight displacement of the adjacent incisors. This regeneration phenomenon was enhanced by the mixture of hydroxyapatite nanoparticles (NPs) in the hydrogel (Eltawila et al., 2021).

#### 2.1.2 Hyaluronic Acid

Hyaluronic acid (HyA), also known as hyaluronan, is a natural polysaccharide found in the body with excellent biocompatibility, biodegradability, and gelling features due to its ability to rapidly bind to water. Some aqueous formulations based on HyA or its derivatives can be injected and subsequently gelate *in situ*, according to their molecular weight and local microenviroment. HyA can cross-link or conjugate with various biomacromolecules and efficiently load various drugs, even nanoparticles. As such, it is widely used in biomedical applications, and has high potential as a controlled drug-delivery material (Kartika et al., 2021; Zhang et al., 2022). The cytocompatibility of HyA has been confirmed in an *in vitro* study using human dermal fibroblasts. After autoclaving, the chemical derivatization of HyA with poly lactic acid retains its chemical structure of the starting copolymers, rheological characteristics, and drug-release properties (Pitarresi et al., 2013). The rheological features ensure that hydrogel is mostly retained on the roughness titanium prosthesis surface, despite the intense shear stress experienced during insertion (Pitarresi et al., 2013; Drago et al., 2014). These illustrate its potential use in orthopedics, especially for the antimicrobial coatings of orthopedic devices for the prevention and/or treatment of OM (Boot et al., 2017; Boot et al., 2020).

Disposable Antibacterial Coating (DAC^®^) (Novagenit Srl, Mezzolombardo, Italy) hydrogel consists of hyaluronan and poly D, L-lactide via covalent linkage. It is used as a disposable, rapidly bioresorbable antimicrobial coating for orthopedic implants. In 2014, a study addressed the following concerns regarding this HyA hydrogel: 1) Can it be used for the controlled release of antimicrobial agents *in vitro*? 2) Can this hydrogel (alone or antimicrobial-laden) coating reduce pathogen colonization on implants? And 3) what is the feasibility of intraoperative manufacture of coating and its resistance to the intense stress generated during the insertion? (Drago et al., 2014). The authors showed that all tested antibacterial compounds (gentamicin, vancomycin, amikacin, tobramycin, N-acetylcysteine, and sodium salicylate) were completely released in less than 96 h. The drug-laden DAC^®^ hydrogel presented bactericidal and antibiofilm effects *in vitro* [against MRSA, methicillin resistant *Staphylococcus epidermidis* (MRSE), *E. coli*, *Pseudomonas aeruginosa*, *Acinetobacter baumannii,* and vancomycin-resistant *Enterococcus faecalis*]. Intraoperative preparation of hydrogel prosthesis coating is feasible. Almost 80% of the hydrogels are retained on the surface of the implants, after the press-fit insertion of the coated implants in rabbit tibias or human femurs (Drago et al., 2014). *In vivo*, loaded or unloaded with 2% (w/v) vancomycin, the hydrogel coating on titanium rods implanted into rabbit tibias had no effect on the volume or timing of bone apposition. In addition, no inflammation was observed (Boot et al., 2017). Meanwhile, Vancomycin-laden DAC^®^ reduced the local bacterial burden (MRAS or MRSE) with more bone implant contact (Lovati et al., 2016; Boot et al., 2020). Recently, a multi-center matched case–control study revealed no infection or adverse events (0/43) in a mega-prosthesis treatment group with antimicrobial-laden DAC^®^ hydrogel coating, compared with the infection rate of 14% (6/43) in a control group without coating, at a mean follow-up of 2 years (Zoccali et al., 2021). Those findings suggest that this treatment strategy could be used safely to prevent early surgical site infections of arthroplasty.

HyA-poly (*N*-isopropylacrylamide) (HA-pNipam) hydrogel is another common delivery system used in OM research. Gentamicin and vancomycin can be loaded into this thermo-responsive HyA-based hydrogel, and are subsequently released in an initial burst followed by a more sustained release pattern. The drugs could be detected for more than 14 days *in vitro* and 10 days *in vivo* (Boot et al., 2021; Foster et al., 2021). This sustained antibiotic-release system markedly increases treatment efficacy (Ter Boo et al., 2016; Ter Boo et al., 2018; Vallejo Diaz et al., 2020; Boot et al., 2021; Foster et al., 2021) without hepatorenal toxicity (Foster et al., 2021).

#### 2.1.3 Chitosan

Chitosan (CS), another polysaccharide biopolymer produced by chitin deacetylation, mainly exists in the shells of crustaceans, insects, and fungal cell walls (Bakshi et al., 2020). Multiple special properties of CS have been reported, including biocompatibility, biodegradability, bioadhesion, low toxicity and immunogenicity, plasticity, modifiability, and printability (Tao F. et al., 2020; Tao et al., 2021). The polycationic nature allows CS to interact with the polyanionic molecules on the bacterial surface to alter bacterial permeability (Raafat et al., 2008). In addition, active amino groups in CS can disrupt RNA and protein synthesis by interfering with DNA (Tao et al., 2021). These molecular structure-related peculiarities attribute to the broad spectrum antibacterial properties of CS (Tan et al., 2013).

Due to the special biological and physicochemical characteristics of CS, CS-based biomaterials are used to simulate the natural extracellular matrix (ECM) and as a drug vehicle. CS has attracted considerable attention in bone tissue engineering, owing to its innate antimicrobial, osteo-inductive, and mechanical properties. CS-based topical drug delivery systems can promote bone regeneration and treat bone diseases, with CS showing promising efficacy in OM treatment (Kazimierczak et al., 2019; Kimna et al., 2019; Tao J. et al., 2020). In addition to antibiotics (Li et al., 2020a; Tao J. et al., 2020), silver can also be released in a sustained manner from CS hydrogel *in vivo* (Croes et al., 2018).

Other natural polymers such as gelatin and alginate (ALG), which have also been studied *in vivo* as vehicles for local drug delivery for the treatment of OM (Wu et al., 2013; Aldrich et al., 2019; Cobb et al., 2019; Sun et al., 2021) (Supplementary Table S1), have been illuminated previously (You et al., 2017; Pedroza-Gonzalez et al., 2021). Although the cell behavior and tissue formation can be significantly affected by changing the properties (such as viscosity and stiffness) of the natural polymers (Daly et al., 2016), the local antibiotic release (including release amount and rate) of these polymers remains unsatisfactory (Wang and Tang, 2019). Some techniques have been designed to control release kinetics, degradation rates, predictability of behavior and quality, and mechanical properties. Synthetic polymers are one such approach.

### 2.2 Synthetic Polymers

PEG-based hydrogels are common synthetic local delivery systems for OM used in *in vivo* studies. Mixing of hydrogels with crosslinked starch (CSt) can inhibit PEG-based hydrogel swelling, thereby reducing the rate of drug release. Covered by a PEG-poly (lactic-co-caprolactone) (PEG-PLCL) membrane, this PEG-based hydrogel coating system allows a sustained vancomycin release, without initial burst release, for approximately 3 weeks *in vitro* and over 4 weeks *in vivo*, and demonstrates a promising antimicrobial activity against *S. aureus*. Additionally, drug loading and the coating thickness regulate the release profile of the hydrogel coating (Li et al., 2017).

Another PEG-based synthetic polymer, four-arm PEG macromers (PEG-4MAL), functionalized with terminal maleimide groups that react specifically with thiols, are functionalized with cell adhesive peptides and cross-linked into a network using thiolated molecules such as protease-degradable peptides with terminal cysteines. The physically encapsulated drugs are released as the hydrogel is degraded, or *via* direct diffusion. This implies that the release of its payload can be tuned with respect to the local microenvironment. Hydrogel-mediated lysostaphin and bacteriophage delivery systems have been shown to eliminate MRSA and *P. aeruginosa* infection in mice models of OM (Johnson et al., 2018; Johnson et al., 2019; Wroe et al., 2020), and release bone morphogenetic protein 2 (BMP-2) in a sustained pattern as well (Johnson et al., 2019). BMP-2-laden PEG-4MAL demonstrated higher osteo-regeneration ability compared to BMP-2-soaked collagen sponges (Shekaran et al., 2014). Moreover, the degradation products of PEG-4MAL hydrogel are excreted via the urine with low toxicity (Johnson et al., 2018).

## 3 Agents

### 3.1 Antimicrobial Agents

Antimicrobial agents are essential for the treatment of OM. Different agents based on hydrogel drug delivery systems have been investigated *in vivo* (Supplementary Table S1).

#### 3.1.1 Antibiotics

Unlike ALBC, using hydrogels as drug carriers greatly expands the selection of antibiotics. Although these drugs can decrease the lower critical solution temperature (LCST) of thermo-responsive hydrogels, such as the HA-pNipam hydrogel, known as the Hoffmeister effect (Ter Boo et al., 2016), the drug-loaded hydrogel remains injectable and gellable, and its payload delivery and applicability are not impacted. Therefore, it is possible to load a wide spectrum of antimicrobial agents into hydrogels (Boot et al., 2021). However, in *in vivo* studies on OM, the most common antibiotics loaded in the hydrogels are gentamicin and vancomycin.

Depending on the different vehicle system designs, gentamicin and vancomycin, the most effective antibiotics against OM, can be released in different patterns (burst, sustained, and burst and sustained). For example, the direct release of vancomycin can be disrupted because its positive charge can interact with the negatively charged ALG chains (Jung et al., 2019). *In vitro*, the duration of antibiotic-release has been shown to last from less than 96 h to more than 6 weeks (Drago et al., 2014; Ter Boo et al., 2016; Li et al., 2017; Jung et al., 2019; Li et al., 2020a; Boot et al., 2021). However, the release time *in vivo* has rarely been reported, but the available data suggest a range of 72 h to more than 6 weeks (Changez et al., 2005; Li et al., 2017; Overstreet et al., 2019; Li et al., 2020a; Foster et al., 2021). After covering with a PEG-PLCL membrane, the CSt-mixed PEG-based vehicle system, coated on a Ti implant by covalently bound, sustainedly released vancomycin for over 4 weeks *in vivo*, without an initial burst release, thereby showing good antimicrobial activity against *S. aureus* (Li et al., 2017). Foster et al. (2021) reported that antibiotics can be released from HA-pNipam hydrogel for over 10 days, with local concentrations higher than the MIC. Increasing the concentration of gentamicin or vancomycin to 44% (w/w) in a composite hydrogel, which consisted of poly (acrylic acid) and gelatin, maintains the local antibiotics concentration at levels higher than MIC for more than 6 weeks (Changez et al., 2005).

*In vivo* use of other antibiotics, such as colistin, tobramycin, daptomycin, and isoniazid, in combination with hydrogels has been reported only sporadically (Spicer et al., 2013; Aldrich et al., 2019; Liu et al., 2019; Overstreet et al., 2019). Recently, daunorubicin, ketoconazole, rifapentine, and sitafloxacin have been demonstrated bactericidal activity against *S. aureus* SCVs. Sitafloxacin can also be used to eliminate methicillin-susceptible and -resistant *S. aureus*, as well as *S. aureus* within an established biofilm (Trombetta et al., 2018). Designing optimal hydrogel delivery systems for these drugs will offer novel treatment strategies for patients with OM.

The widespread emergence of antibiotic-resistant bacteria, which has heralded in a post-antibiotic era (Roca et al., 2015), has prompted the development of strategies other than traditional antibiotic therapy. Some antimicrobial agents, such as bacteriophages, antimicrobial peptides (AMPs), nitric oxide (NO), and silver (Ag), have been applied to the hydrogel local drug delivery system; therefore, these strategies open new avenues for the treatment of OM (Table 1; Supplementary Table S1).

**TABLE 1:** Summary of advantages and limitations of different agents in hydrogels for OM.

Agents		Advantages	Limitations	References
Antimicrobial agents	Antibiotics	A wide variety of selection; broad clinical application; easy to manipulate	Resistance species	Overstreet et al. (2019), Eltawila et al. (2021), Foster et al. (2021), Sun et al. (2021)
	AMPs	A wide variety of selection; no resistance	Cytotoxicity	Guoli Yang et al. (2018)
	Lysostaphin	Highly specific anti-staphylococcal activity; synergistic effects with β-lactam antibiotics	Narrow antibacterial spectrum; the development of neutralizing antibodies; resistance species	Johnson et al. (2018), Johnson et al. (2019), Johnson et al. (2019)
	NO	Multifunctional (antibacterial, promotion of osteogenic differentiation, inflammation regulation)	The NO release is irreversible and difficult to be controlled	Li et al. (2020b)
	Bacteriophages	Extremely pervasive, sustained antimicrobial effect; synergistic effects antibiotics	Resistance species	Cobb et al. (2019), Ferry et al. (2020), Wroe et al. (2020)
	MΦs	MΦs are presumed to mediate biofilm clearance, which transform the bacteria from the dormant state into the active planktonic state, sensitizing them to antibiotics	Limited half-life; no prophylactic efficacy	Aldrich et al. (2019)
	Sliver	Being widely used in clinic	The efficacy and safety remain controversial in orthopedic application	Croes et al. (2018), Xu et al. (2018)
BMP-2		Osteo-generation effect; co-delivery with other agents	—	Aldrich et al. (2019), Johnson et al. (2019)
PDA		Multifunctional (good adhesion and reducing capability to deposit bioactive molecules and synthesize antimicrobial agents, inducing the mineralization of hydroxyapatite, excellent photothermal properties)	—	Xu et al. (2018), Li et al. (2020b)

AMP, Antimicrobial Peptide; MΦs, Macrophages; BMP, Bone Morphogenetic Protein; OM, Osteomyelitis; —, not mentioned.

#### 3.1.2 Antimicrobial Peptides

AMPs, usually short positively charged peptide sequences (Ter Boo et al., 2015), target a specific features of microbial cell membranes, which distinguishes the broad range of pathogen species from multicellular plants and animals (Zasloff, 2002). The amphipathic structure of cationic AMPs confers their ability to bind to and disturb the pathogen cellular membrane, and induce the cell death (Epand and Vogel, 1999). In contrast to antibiotics, it is not possible to develop acquired resistance toward AMPs for sensitive bacterial strains, despite the inherent resistance in some bacterial species (Zasloff, 2002). The use of AMPs in OM is quite limited. Yang G. et al. (2018) used RADA16 to form a stable hydrogel scaffold that controlled the release of Tet213, a kind of cationic AMPs. The release of Tet213 was sustained for up to 28 days. *In vitro*, the growth of *S. aureus* was inhibited by this AMPs-laden hydrogel, while the proliferation of bone mesenchymal stem cells (BMSCs) was promoted. *In vivo*, RADA16-AMP self-assembling peptide has demonstrated a promising effect on bone formation.

#### 3.1.3 Lysostaphin

Lysostaphin is a metallo-endopeptidase produced by *S. simulans* (Schindler and Schuhardt, 1964). The bacteriolytic enzyme exhibits a highly specific anti-staphylococcal activity (Kumar, 2008). Notably, this bacteriolytic enzyme, which possesses high anti-staphylococcal activity, exhibits antimicrobial activity against resistant strains, including MRSA, vancomycin-intermediate *S. aureus*, vancomycin resistant *S. aureus* and *S. epidermidis* (Climo et al., 1998; Patron et al., 1999; Mohamed et al., 2014). In contrast to most small-molecule antibiotics, the antimicrobial activity of lysostaphin is not dependent on the bacterial metabolic state; hence, it is active against bacteria in biofilms (Patron et al., 1999), and effectively kills bacteria at lower concentrations (Wu et al., 2003). Furthermore, lysostaphin does not impact the osteogenic differentiation of human cells (Johnson et al., 2018). Unlike systemic use of antibiotics, the species-specific nature of lysostaphin does not perturb the gut microbiota. These features make lysostaphin an potential candidate for the treatment of staphylococcal OM.

Johnson et al. (2018) engineered a low-toxicity, injectable PEG-4MAL hydrogel. The elastic and adhesive features of the hydrogel were not affected by the addition of lysostaphin; meanwhile, the release of lysostaphin could be controlled by tuning the mesh structure of the hydrogel. Also, due to the protease-degradable peptide cross-linked in the hydrogel, lysostaphin release is influenced by local protease activity, which is elevated in the inflammatory microenvironment triggered by bacterial infection (Wolcott et al., 2008). This design maintains the activity of lysostaphin over 14 days (Johnson et al., 2018; Johnson et al., 2019). In a murine model, the lysostaphin-loaden PEG-4MAL hydrogel was shown to clear the infections and supported fracture cure or defect repair (Johnson et al., 2018; Johnson et al., 2019). Seven days post-infection, a multiplexed cytokine array assay revealed a sterile state had been restored in mice in the lysostaphin-laden hydrogel group, and after 5 weeks, bacterial counts revealed persistent infection in mice in the control group; however, animals in the trial group remained sterile, confirming the results at 1-week post-surgery. The group treated with the sustained lysostaphin topical release system showed comparable bone regeneration and mechanical properties to the uninfected group. Notably, sustained release of lysostaphin from hydrogel presented efficient anti-biofilm activity, while the soluble lysostaphin (no hydrogel) did not.

Despite this, there remain two concerns regarding the use of lysostaphin namely the development of neutralizing antibodies (Johnson et al., 2018; Johnson et al., 2019), and the potential resistance to the enzyme (Boyle-Vavra et al., 2001; Climo et al., 2001). Interestingly, lysostaphin synergizes with β-lactam antibiotics, and lysostaphin can render the resistant strains susceptible to the antibiotics (Climo et al., 2001; Kiri et al., 2002). The *in vivo* effects of the combined delivery of lysostaphin and antibiotics warrant further investigations.

#### 3.1.4 Nitric Oxide

NO exerts various important physiological functions. In addition to its role as a signaling molecule, NO is involved in immune regulation and wound healing (Hoang Thi et al., 2018; Li et al., 2020b). Low concentrations of exogenous NO can enter pathogens and provide a degree of antibacterial activity. Additionally, upon reaction with superoxide (·O2−), NO produces peroxynitrite (·ONOO−), which possesses tremendous virulence against bacteria. However, the NO release is irreversible and difficult to be controlled (Yang T. et al., 2018).

**FIGURE 2:**
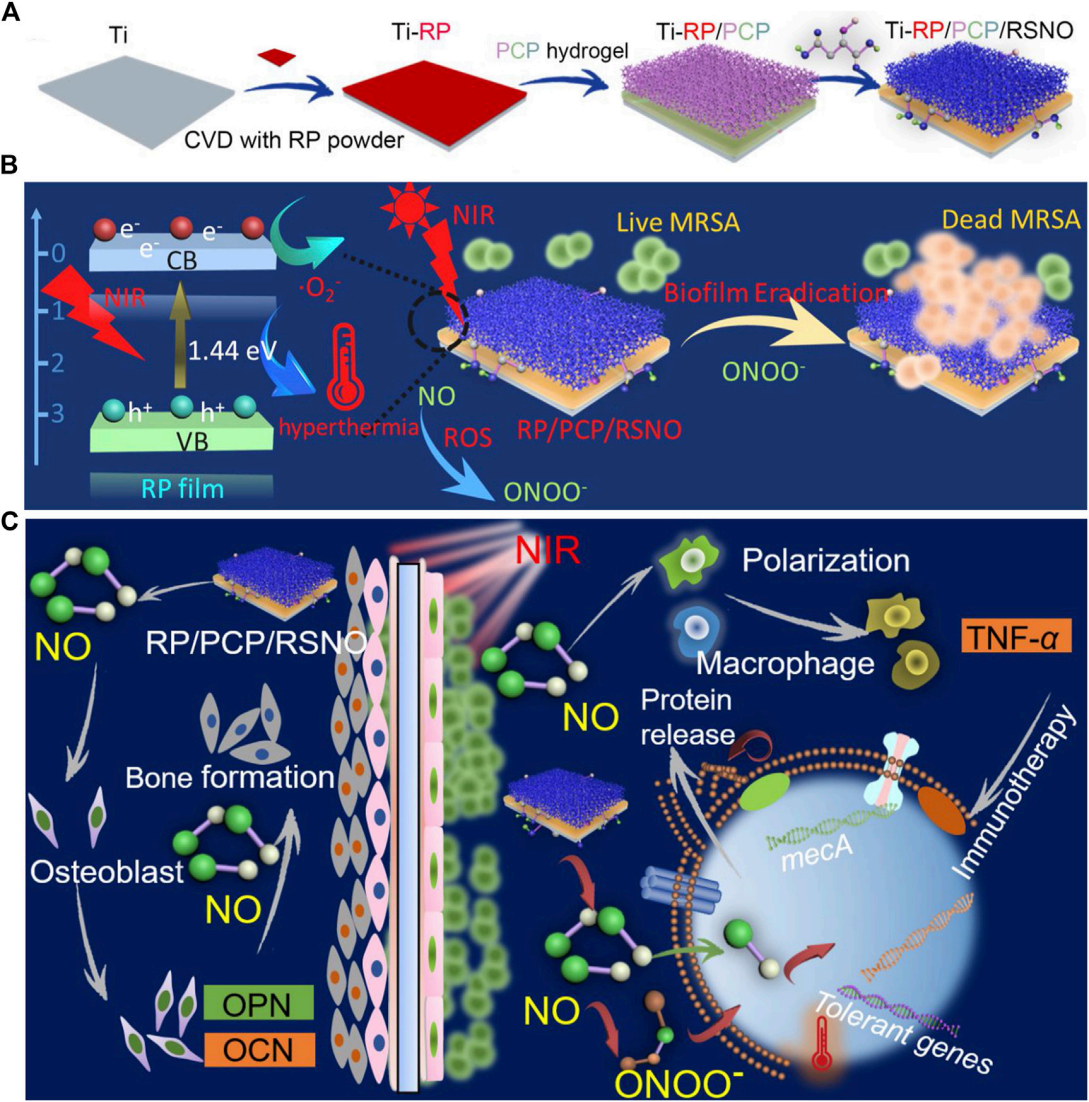
**(A)** Schematic representation of the Ti-RP/PCP/RSNO hydrogel coating system preparation process. **(B)** Schematic representation of NIR triggered biofilm eradication. **(C)** Schematic representation of the mechanism of promoted bone formation and MRSA biofilm eradication. Ti, titanium; CVD, chemical vapor deposition; RP, red phosphorous; PCP, polyvinyl alcohol hydrogel modified with chitosan and polydopamine; RSNO, NO donor of S-nitrosuccinic acid; NIR, near-infrared; MRSA, methicillin-resistant *Staphylococcus aureus*; NO, Nitric oxide. Reprinted with permission from Li et al. (2020b). Copyright: 2020 American Chemical Society.

To use NO more effectively, Li et al. (2020b) have engineered a hydrogel system, PCP/RSNO, comprising a polyvinyl alcohol (PVA) hydrogel modified with CS, polydopamine (PDA), and a NO-release donor (Figure 2). By coating the hydrogel with a red phosphorous (RP) nanofilm, deposited on a titanium implant (Ti-RP/PCP/RSNO), the release of NO and ·O2− could be controlled using near-infrared (NIR) light, which subsequently formed ·ONOO-. Synergism between ·ONOO−, ·O2−, and hyperthermia at 808 nm NIR irradiation destroyed over 93.1% of an MRSA biofilm *in vitro*, demonstrating greater effectiveness than vancomycin (76.2%). Furthermore, the anti-biofilm efficiency was approximately 91.9%. In addition to the antibacterial mechanism against MRSA biofilms, the released NO promoted the osteogenic differentiation and regulated inflammatory polarization by upregulating the expression of *Alp*, *Opn*, *Ocn*, and *Tnf-*α. The efficient biofilm eradication (99.2%) and bone formation induced by NO released from this coating system under NIR irradiation have also been confirmed *in vivo*.

#### 3.1.5 Bacteriophages

Bacteriophages exert a sustained antimicrobial effect and have potential to prevent and control bacterial biofilms (Sarker et al., 2012; Geredew Kifelew et al., 2019). Bacteriophages are associated with most known species of bacteria. The high specificity prevents side effects associated with the human microbiome (Rhoads et al., 2009; Petrovic Fabijan et al., 2020). Recently, a clinical trial reported the safety and tolerability of adjunctive bacteriophage approach for the treatment of severe *S. aureus* infection (Petrovic Fabijan et al., 2020).

Wroe et al. (2020) have engineered an injectable PEG-4MAL based hydrogel that encapsulate and delivered *P. aeruginosa* bacteriophages to the site of OM. Bacteriophages were released in a controlled manner with a retained bacteriolytic activity. *In vitro*, both planktonic and biofilm pathogens were effectively eliminated using this phage delivery system. Furthermore, the metabolic activity of human mesenchymal stromal cells was not disturbed. Seven-days following implantation in murine radial infectious defects, live *P. aeruginosa* counts were reduced 4.7-fold in the bacteriophage-laden hydrogel group, compared with that in the bacteriophage-free hydrogel group.

Ferry et al. (2020) reported a case of recurrent *S. aureus* knee mega-prosthesis infection treated with a bacteriophage-laden DAC^®^ hydrogel. Unfortunately, the patient suffered a myocardial infarction and underwent emergency stenting and received dual antiplatelet therapy 5-days later. Consequently, bleeding persisted at the surgical site and led to another prosthesis exposition for debridement. No *S. aureus* was found in culture, although three other bacteria strains were identified.

These results support the development of bacteriophage-delivery hydrogels, and provide new therapeutic frontiers for OM. To improve the safety of phage therapy, Cobb et al. (2019) modified the bacteriophages with CRISPR-Cas9 to remove all staphylococcal cytotoxin and enterotoxin genes, thereby preventing toxin contamination in the phage solution. However, the phage-delivery alginate hydrogel was efficacious against soft tissue infections *in vivo*, but not for bone infections.

Resistance is another obstacle hindering the use of bacteriophages. Phages depend on specific proteins to adhere to and infect bacteria. Thus, it is possible to develop resistance to phages, like antibiotics (Yilmaz et al., 2013). Although this might be limited due to the coevolution of bacteriophages and their hosts (Sweere et al., 2019). The co-delivery of bacteriophages and antibiotics could be a potential approach. Synergism upon dissolving the biofilm of MRSA and *P. aeruginosa* has been demonstrated (Yilmaz et al., 2013).

#### 3.1.6 Macrophages

**FIGURE 3:**
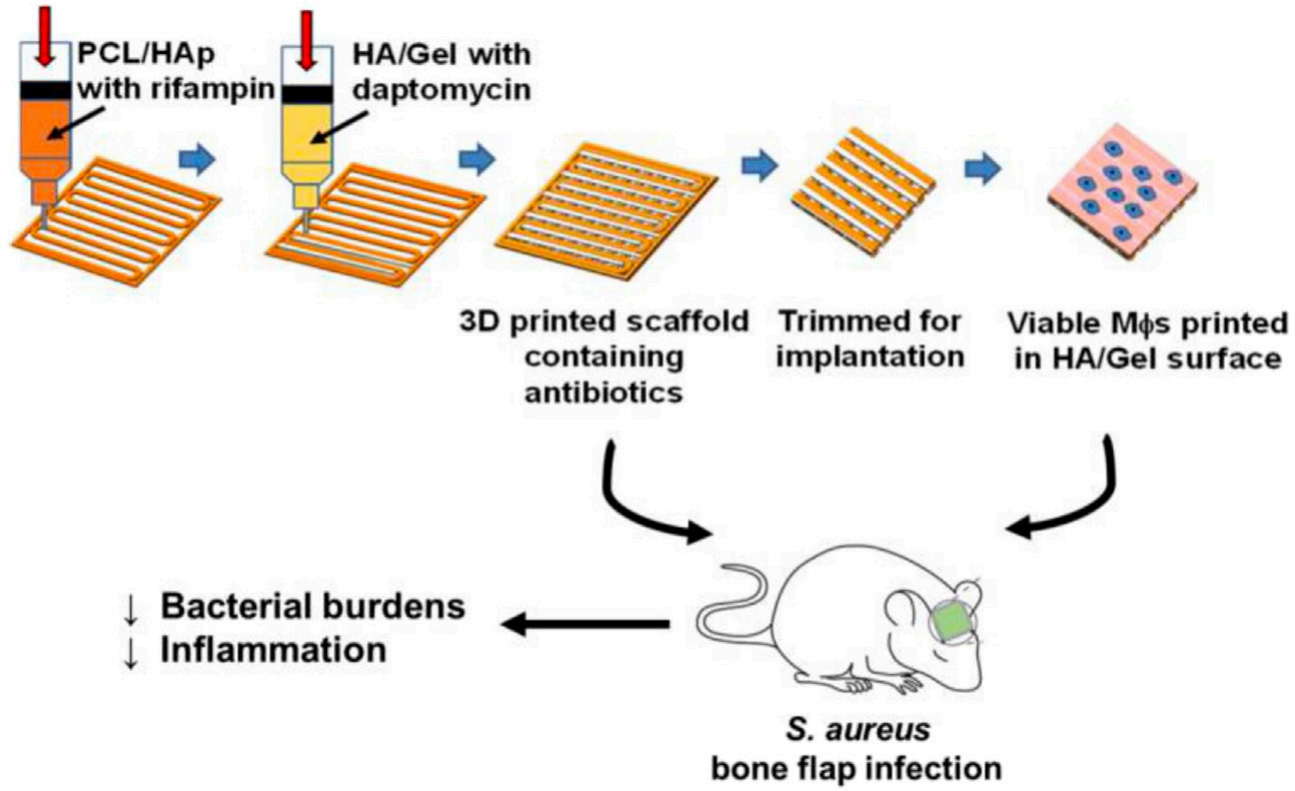
Schematic representation of 3D bioprinted scaffolds containing viable macrophages and antibiotics, and reduction of bacterial burdens in a mouse model of *S. aureus* craniotomy-associated biofilm infection. PCL, polycaprolactone; HAp, hydroxyapatite; HA, hyaluronic acid. Reprinted with permission from Aldrich et al. (2019). Copyright: 2019 American Chemical Society.

Activated macrophages (MΦs) significantly limit *S. aureus* biofilm growth and colonization (Hanke et al., 2013). It is also presumed that activated MΦs facilitate biofilm clearance, which transform the dormant bacteria within the biofilm into a metabolically active planktonic state and sensitive to antibiotics (Hanke et al., 2013; Aldrich et al., 2019). In a proof-of-concept study, Aldrich et al. (2019) demonstrated the synergistic effects of MΦs and antibiotics in a 3D bio-printed antibiotic-loaded bone scaffold, which promoted *S. aureus* clearance in a craniotomy-associated infection in mice (Figure 3). For treatment, the scaffolds were placed in the defect area at day 7 post-infection, and the incorporation of MΦs further reduced bacterial burden compared to antibiotics alone. The exact antimicrobial mechanism remains to be determined. It is possible that the direct antimicrobial effect of MΦs, and cytokines/chemokines secreted by activated MΦs enhanced the antibacterial activity of other glia/leukocytes. However, the synergistic effects were detected only in the treatment paradigm, but not in the prevention paradigm in which the scaffolds were inserted 1 day prior to *S. aureus* inoculation. One possibility reason is lack of some signals, which are present in an established biofilm, to activate MΦs. Meanwhile, the authors found that the beneficial effect of MΦs was transient. No more decrease of bacterial titers was observed 7 days after treatment. This was likely attributed to the limited half-life of MΦs when exposed to the large number of bacteria in the biofilm that produced lytic toxins. Although the authors observed bacterial burden only in Galea and Brain, but not in bone tissue. The study presents an immune-based 3D bioprinting approach to promote biofilm clearance.

#### 3.1.7 Silver

Before the discovery of penicillin, silver (Ag) was used clinically as a bactericidal agent (Chopra, 2007). Due to the nucleophilic functions of proteins, enzymes, and cell membrane components in bacteria, they react with Ag cations (Ag^+^), which then disrupt their function and displace metal ions, such as Zn^2+^ and Ca^2+^. These subsequently induce the bacterial death (Hetrick and Schoenfisch, 2006). Silver nanoparticles (AgNP) can be synthesized *in situ* using polydopamine (PDA) and mineralized on PEG diacrylate (PEGda) hydrogels. Therefore, the so-called AgNPs/PDA-coated PEGda hydrogels were designed for the treatment of anti-infection (Xu et al., 2018). Controlled Ag delivery inhibits the growth of *S. aureus* and *E. coli*; *in vivo*, the AgNP/PDA gel efficiently repaired maxillary defects without infection.

To our knowledge, Ag-loaded hydrogels have been rarely studied for the treatment of OM. Croes et al. (2018) have developed a CS-based coating with AgNPs, which showed burst and sustained drug release properties. However, no antimicrobial effects were found *in vivo*. Furthermore, radiological signs of aggravated OM were observed. The authors attributed the poor antibacterial property to the cytotoxicity for neutrophils at antimicrobial Ag concentrations, and the diminished phagocytic effect at nontoxic concentrations. In orthopedic applications, the true efficacy and safety of Ag remains controversial (Prokopovich et al., 2013; Wafa et al., 2015; Masters et al., 2019). Future research should investigate the ideal formulation and concentration of Ag or AgNPs for safe clinical use.

### 3.2 Bone Morphogenetic Protein 2

Recombinant BMPs have been developed to address the challenge of segmental bone defects. BMP-2 promotes cell proliferation, alkaline phosphatase activity, differentiation, and mineralization *in vitro* and *in vivo* (Tao et al., 2021). The US FDA has approved the use of BMP-2 to facilitate bone formation (Burkus et al., 2002). However, its use is limited by adverse events associated with supraphysiological doses, including inflammation and heterotopic bone formation (Hustedt and Blizzard, 2014). Carriers with controlled BMP-2 release have been developed to promote bone regeneration and reduce the incidence of adverse effects (Hustedt and Blizzard, 2014; Shekaran et al., 2014; Huang et al., 2017; Han et al., 2020; Sun et al., 2020; Tao et al., 2021). A BMP-2-loaded PEG-4MAL hydrogel has been shown to generate better quality bone compared to a BMP-2-laden collagen sponge (Shekaran et al., 2014).

Interventions that promote bone growth while fighting bone infections have the potential to significantly reduce the incidence of non-union and improve patient prognosis. BMP-2 encapsulated in hydrogels with antibiotics can be released in an initial burst manner followed by a sustained release pattern (Jung et al., 2019). Previous studies demonstrated that vancomycin incorporated into the hydrogels did not significantly interfere with the release of BMP-2 (Jung et al., 2019), and that the loaded antibiotics, including ampicillin, cefazolin, dibekacin, vancomycin, teicoplanin, and minocycline, did not inhibit the ability of rhBMP-2 to repair cranial defects (Suzuki et al., 2006). The sustained release of BMP-2 led to prolonged activity, and resulted in effective osteogenic differentiation and bone reconstruction (Kim et al., 2018; Jung et al., 2019). *In vivo*, BMP-2 and antibiotic-co-encapsulated hydrogel group increased bone reconstruction (Suzuki et al., 2006) and significantly improved biomechanical features compared to the other groups (Suzuki et al., 2006; Jung et al., 2019). The rationale for this strategy was that the co-administration of antibiotics and BMP-2 preserved their own functionality. Moreover, co-administration showed more effective suppression of bacteria compared to the antibiotic alone; this result could be attributed to the rapid proliferation of bone marrow stromal cells induced by BMP-2 outpacing the infectivity and proliferation of pathogen (Jung et al., 2019).

In a study investigating a bifunctional hydrogel, Johnson et al. (2019) also showed that BMP-2 and lysostaphin-co-encapsulated PEG-4MAL hydrogels prevented *S. aureus* infection and promoted segmental bone defect regeneration. The co-encapsulated hydrogel with *S. aureus* group showed equal amounts of new bone formation as the sterile hydrogel alone group.

### 3.3 Polydopamine

Due to its excellent biocompatibility, hydrophilicity and adhesion reactions with various molecules, PDA has been used pervasively in tissue engineering. In addition to being a crosslinking agent, PDA has been shown to induce the mineralization of hydroxyapatite on demineralized dentin (Zhou et al., 2012), and presents good adhesion and reducing capabilities to deposit bioactive molecules (Sileika et al., 2011; Xu et al., 2018).

PDA has been used to synthesize AgNPs *in situ* on a PEGda scaffold to construct an AgNPs/PDA-coated PEGda hydrogel (Xu et al., 2018). This hydrogel delivery system exhibited excellent cytocompatibility with strong antimicrobial effects against *S. aureus* and *E. coli*, and simultaneously promoted bone generation, due to dual functions of anti-bacterial activity of AgNPs and graft mineralization of PDA. The expression of some osteogenic genes was upregulated *in vitro*, including osteocalcin, runt-related transcription factor 2, bone sialoprotein, and alkaline phosphatase. The rat maxillary bone defects were efficiently repaired (Xu et al., 2018).

In addition, PDA present excellent photothermal properties. NIR irradiation activates photothermal PDA in the hydrogel, and local hyperthermia destroies the integrity of bacteria, leading to bacterial inactivation in a synergistic manner (Gao et al., 2019; Li et al., 2020b). In the Ti-RP/PCP/RSNO system, PDA in the hydrogel increases the bonding strength between the hydrogel and RP-modified implants (Li et al., 2020b). Meanwhile, NIR irradiation activated the photothermal effect of PDA. The generated local hyperthermia disrupted bacterial integrity and eliminated the MRSA burden in a synergistic manner with the simultaneous generation of ·ONOO− and ·O2−.

## 4 Methods to Control Drug Release

### 4.1 Extending Drug Release

Treatment duration is an important factor affecting the success of antibiotic therapy for OM. Thus, in addition to mechanical strength, degradability and histocompatibility, drug elution kinetics are important for vehicle choice. For most hydrogels, the loaded drugs are released in a burst manner, driven by diffusion. *In vitro*, >90% of antibiotics are released within the first day, while the remaining are released within the following 4 days. However, *in vivo*, the release time may be extended due to the potentially limited fluid volume and mass transfer at the infectious site (Ruan et al., 2016; Li et al., 2017; Haider et al., 2018; Johnson et al., 2018). Extending or controlling the effective duration of drug release can be achieved by successive injections (Boot et al., 2021) or modification of the hydrogel (Sun et al., 2021).

#### 4.1.1 Composite Materials

The development of composite materials can overcome the shortcomings of the individual constituents. The HA-pNipam hydrogel and CSt-mixed PEG hydrogels are composite polymers derived from natural and synthetic materials, respectively. Gentamicin and vancomycin loaded in the HA-pNipam hydrogel were released for more than 336 h *in vitro* and 10 days *in vivo* (Boot et al., 2021; Foster et al., 2021). Drugs release was delayed in a PEG-based hydrogel mixed with CSt, to 3 weeks *in vitro* and 4 weeks *in vivo* without an initial burst release (Li et al., 2017).

An *in situ* gelling alginate/HyA hydrogel, designed by Jung et al. (2019), continuously released both vancomycin and BMP-2 for 6 weeks *in vitro* without significant cytotoxicity. The results of the *in vivo* study demonstrated that this vancomycin/BMP-2-laden-alginate/HyA hydrogel could efficiently inhibit *S. aureus* proliferation and promote bone regeneration. Another example of a PEG composites, a triblock PLA-DX-PEG hydrogel (PDLLA-p-dioxane-PEG) composed of PLA:DX:PEG at a molar ratio of 5:1:3. *In vitro*, almost 40% of the laden teicoplanin was released within the first 24 h, and the concentrations above the MIC 90% for *S. aureus* were maintained for 2 weeks (Suzuki et al., 2006).

#### 4.1.2 Transglutaminase

Transglutaminase (TGase) can produce conjugates derivatized at the level of Gln and/or Lys residues, and has been used successfully in various biotechnological applications (Duarte et al., 2020; Dell'Olmo et al., 2021; Sun et al., 2021). The hydrogels, crosslinked by TGase, can control the release of loaded drugs at the targeted site, acting as “smart” delivery systems. In a novel vancomycin-impregnated gelatin/alginate hydrogel crosslinked by TGase, over 90% crosslinking was achieved (Sun et al., 2021). The release time of the encapsulated antibiotics increased with increasing TGase concentrations, from approximately 20 min without TGase to more than 120 h with 1% TGase. In an *in vivo* study of implant-associated infection in rat, the vancomycin-treated group showed reduced biofilm formation and inflammation, and significant bone regeneration, even after inoculation with a high dose of MRSA. Notably, these beneficial effects were observed with the vancomycin-laden hydrogel, but not with the gentamicin-loaded hydrogel.

#### 4.1.3 Nanoparticles

NP-based anti-infection strategies have promising for biomedical applications. This can be attributed to the large surface area to volume ratio and the flexibility in tuning their charactristics (Zhang et al., 2008). Many biocompatible and biodegradable NPs, such as liposomes and polymeric NPs, have been used as vehicles to control antimicrobial delivery (Forier et al., 2014; Ma et al., 2019; Tao J. et al., 2020). Using this approach, the limitations of antibiotic treatment can be overcome, including inefficient drug release, enzymatic inactivation of drugs, and cytotoxicity (Meers et al., 2008). Compared to free drugs, the encapsulated antibiotics can efficiently penetrate extracellular polymeric substances, resulting in the delivery of therapeutic doses at the target site.

CS-based NPs are used as drug vehicles due to their ideal biological characteristics, such as biocompatibility, antimicrobial properties, and low toxicity. Tao J. et al. (2020) hypothesized that the positive charge of quaternary ammonium CS (QCA) and negative charge of carboxylated CS (CC) drive the electrostatic adsorption-driven assembly of NPs, which can efficiently load the water-soluble antibiotics. Based on the hypothesis, they engineered vancomycin-NPs incorporated in CS-gel to construct an injectable thermosensitive vancomycin-NPs/gel drug delivery system. From CS hydrogel alone, vancomycin was released within 1–5 days (Ruan et al., 2016; Haider et al., 2018). In contrast, vancomycin release was extended over 26 days, and 65% of the laden antibiotic was released from the vancomycin-NPs/gel *in vitro*; this meets the clinical demand for OM treatment. In a rabbit tibia *S. aureus* OM model, the sustained release of vancomycin reduced the white blood cell (WBC) count and C-reactive protein (CRP) levels at 4–8 weeks. Radiological and histological analyses showed that the vancomycin-NPs/gel accelerated bone repair under OM conditions (Tao J. et al., 2020).

Liposome-NPs are also commonly applied as drug delivery polymers, since their phospholipid bilayer structure that mimics the cell membrane, enabling fusion with pathogen cell membrane (Allen and Cullis, 2013; Zununi Vahed et al., 2017). Subsequently, the loaded drugs are released to the cellular membrane or cytoplasm of the pathogens (Malam et al., 2009). Studies have demonstrated that encapsulating liposome NPs into hydrogels further extends drug release, since the loaded drugs meet two barriers during release: the liposome and hydrogel networks (O'Neill et al., 2017; Liu et al., 2019). As an excellent vehicle for N′-dodecanoylisonicotinohydrazide (DINH), the drug-laden NPs can be easily prepared and encapsulated into a PLGA-PEG-PLGA hydrogel by simple mixing. The incorporation of liposome NPs does not interfere with the properties of the hydrogel. An *in vivo* pharmacokinetics analysis showed that, compared with the liposome-free hydrogel, a liposome-NPs-hydrogel DINH delivery system released drugs at target sites over a longer duration, with stable drug concentrations. These properties suggest that this system may have potential for localized bone tuberculosis (Liu et al., 2019).

Ag-based NPs have received considerable attention with satisfactory efficacy in wound care. Because the bioavailability and antimicrobial activity of free Ag ions is reduced due to the rapid sequestration by proteins and other cellular components in the wound (Xiu et al., 2011). Despite their sustained release property, AgNPs in the CS gel coating did not show the expected antibacterial efficacy, and even showed signs of aggravating the infection due to its cytotoxicity (Croes et al., 2018). Identifying the ideal formulation and concentration of AgNPs is a theme for future research.

### 4.2 Passive and Active Release

Most sustained drug-release systems prolong the release of agents through passive mechanisms, including diffusion, swelling, and erosion (Ter Boo et al., 2015). Drug diffusion depends on the difference in concentration between the inside and outside of the gel and the length of the diffusion path. Swelling of the hydrogel promotes diffusion of the encapsulated drugs. Bulk erosion of the hydrogel reduces the path length and facilitates drug release with the eroded portion.

Studies have investigated stimulus-sensitive hydrogels, which adapt their physical features and cleave attached chemical groups in response to various stimuli (Li et al., 2020a; Li et al., 2020b; McCarthy et al., 2021). For example, hyaluronidase is an *S. aureus* metabolite related to species spreading (Gu et al., 2015). The acidic nature of *S. aureus* infectious lesions could be enhanced by hyaluronidase activity (Radovic-Moreno et al., 2012; Wang et al., 2017). Therefore, HyA has been selected as a trigger to release agents on demand. In addition to passive release, drugs can be released based on the concentration of bacteria or their metabolites. Li et al. (2020a) developed a HyA-CS/β-glycerophosphate (β-GP)-based thermosensitive hydrogel. Using this intelligent drug-delivery system, which was triggered by HyA, vancomycin hydrochloride was released in a burst or burst-sustained pattern. As the concentration of hyaluronidase increased, the active release pattern was enhanced. *In vitro*, vancomycin concentrations were much higher than the MIC after 25 days, whereas *in vivo*, the drug was completely released and detected for up to 30 days, which was sufficient for preventing or treating infection.

In the smart delivery system designed by Johnson et al. (2018), protease-degradable cross-linking peptide GCRDVPMSMRGGDRCG (VPM) was covalently incorporated into a PEG-4MAL network. In addition to a passive sustained release pattern, due to the inclusion of VPM, the encapsulated drugs could be actively released depending on the local protease levels. These are often elevated in the inflammatory microenvironment triggered by bacterial infection (Wolcott et al., 2008).

## 5 Applications

### 5.1 Implant Coating

**FIGURE 4:**
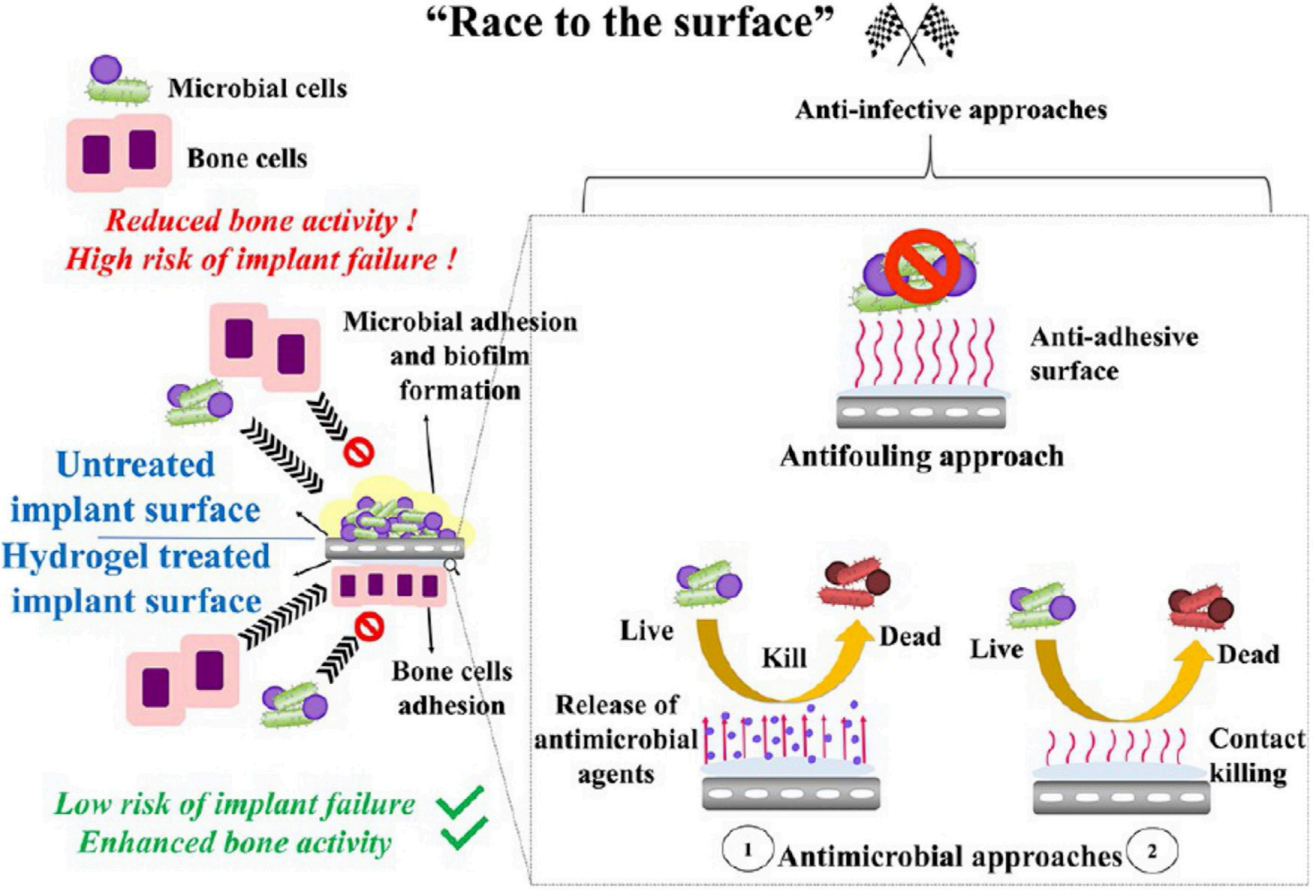
Schematic illustration of the winning “race of the surface” of anti-infective hydrogels. Reprinted with permission from Garg et al. (2021). Copyright: 2021 American Chemical Society.

In addition to filling dead space directly, drug-laden hydrogels are mainly used to coat orthopedic implants (Li et al., 2017; Boot et al., 2020; Li et al., 2020b; Boot et al., 2021; Foster et al., 2021; Sun et al., 2021). Almost 80% of the hydrogel coated on the implants was found to remain after press-fit insertion (Drago et al., 2014). These drug-laden hydrogel coatings have the following advantages: 1) inhibition of bacterial colonization on the implant in the early postoperative phase, to win the “race to the surface” (Figure 4); 2) a favorable safety profile, since high local concentrations and complete controlled drug release over a relatively short period may prevent antibiotic resistance and possible side effects on bone healing; 3) versatility, as different antimicrobial agents can be selected for mixing during the procedure; 4) ease of handling; 5) low costs (Drago et al., 2014). The hydrogel coatings for OM have been primarily studied in rats, rabbits, and sheep, to investigate their prophylactic efficacy (Li et al., 2017; Li et al., 2020b; Boot et al., 2020; Boot et al., 2021; Foster et al., 2021; Sun et al., 2021). The release of agents in a burst and sustained manner reduces the inflammatory response and optimizes the antibacterial effect, demonstrating efficacy towards preventing or treating implant-associated infections.

### 5.2 Scaffolds

An optimal bone graft to treat bone defects induced by infection should possess the following properties: 1) a biphasic drug-release profile with enhanced drug elution kinetics; 2) biodegradability; and 3) osteo-conductivity (Masters et al., 2019). Research is ongoing to improve the properties of bio-scaffolds by loading hydrogels with different frames.

#### 5.2.1 Tricalcium Phosphate Scaffold

Tricalcium phosphate (TCP) is biodegradable, biocompatible, and osteoconductive. TCP cements have inherent porosity and high pore interconnectivity, which enhance the adsorption ability and release of loaded drugs, thus has been used widely as a bone graft (Almirall et al., 2004; Kondo et al., 2005; Silverman et al., 2007; Giavaresi et al., 2012; Wu et al., 2013). However, TCP cements rapidly release the impregnated drugs. Approximately 80% of laden antibiotics are released within 4 days (Teo et al., 2011; Wu et al., 2013).

To engineer an optimal antimicrobial delivery system for the treatment of OM that is easy to handle, biodegradable, and ensures a constant drug release at an effective concentration for a long period, antibiotic-laden hydrogels were introduced into a TCP scaffold (Wu et al., 2013; Li et al., 2020a). Following vacuum adsorption in the hydrogel solution to form the coatings, the TCP scaffold maintained its polyporous structure. This antibiotic delivery system presented elution kinetics characterized by an initial burst followed by zero-order release sustained for more than 25 days *in vitro* and 30 days *in vivo* (Wu et al., 2013; Li et al., 2020a), with higher concentration than the MIC over 25 days *in vivo* (Li et al., 2020a). Following implantation in a rabbit femur condyle OM defect model, the antibacterial and osteogenic effects of these delivery systems were enhanced compared to those of intramuscular antibiotic injection or TCP scaffold alone, which are routine approaches (Wu et al., 2013; Li et al., 2020a).

#### 5.2.2 The 3D-Bioprinting Approach

3D bioprinting has demonstrated excellent potential in bone tissue engineering. Using different strategies, material containing living cells is deposited to fabricate living volumetric constructs in a layer-by-layer manner, such as vascularized bone-like fragment (Byambaa et al., 2017; Ashammakhi et al., 2019). However, studies on the use of 3D bioprinting for OM are rare. Aldrich et al. (2019) have developed a 3D bioprinting bone scaffold engineered for sustained local antibiotic release, in combination with the incorporation of MΦs that possessed potent antimicrobial activity. This 3D-printed scaffold was constructed with methacrylated hyaluronic acid (Me-HyA)- and methacrylated gelatin (Me-Gel)-based hydrogels encapsulating daptomycin between a polycaprolactone/hydroxyapatite frame incorporated with rifampin. MΦs were printed on the surface of the HyA/gel hydrogel matrix in L929 solution to maximize cell viability. In an *S. aureus* craniotomy-associated biofilm mouse model, antibiotic scaffolds with MΦs reduced the bacterial burden (Figure 3); however, infection in the bone defect was not investigated. Due to the short life span of MΦs, this strategy only demonstrated an early therapeutic effect, which was lost at 7 days, with no preventive effects were observed. This proof-of-concept study provides an insight into 3D bioprinting technology in the OM field.

### 5.3 Nanotopography

Stem cell proliferation and differentiation can be modulated by the external microenvironments and specific biophysical cues. As a stream of stem research, nanotography, with diverse biomaterials and different surface geometries, can precisely and efficiently regulate the behavior of stem cells and enhance their abilities through specific cell-surface interaction (Kim et al., 2017; Thomas et al., 2018). Vertically aligned 1D nanomaterials have been used in advanced biomedical applications (Kwak et al., 2015; Poudineh et al., 2018). Living cells and tissues can interface on these efficient platforms. Due to the nanoscale diameters and sharp tips, they can pierce cell membrane, impacting cell viability. The volumes of mammalian and bacterial cells differ. By tuning their physical features (i.e., the relative size of the nanoneedles compared to the cells), these nanomaterials can pierce the pathogen cell membrane without disturbing the integrity of mammalian cell membranes (Mas-Moruno et al., 2019). Furthermore, the long 1D structure facilitates cellular interfacing with the external microenvironments, and the high-aspect-ratio feature of their sharp tip structure effectively prevents biofilm formation via mechanical lysis (Pogodin et al., 2013; May et al., 2016).

**FIGURE 5:**
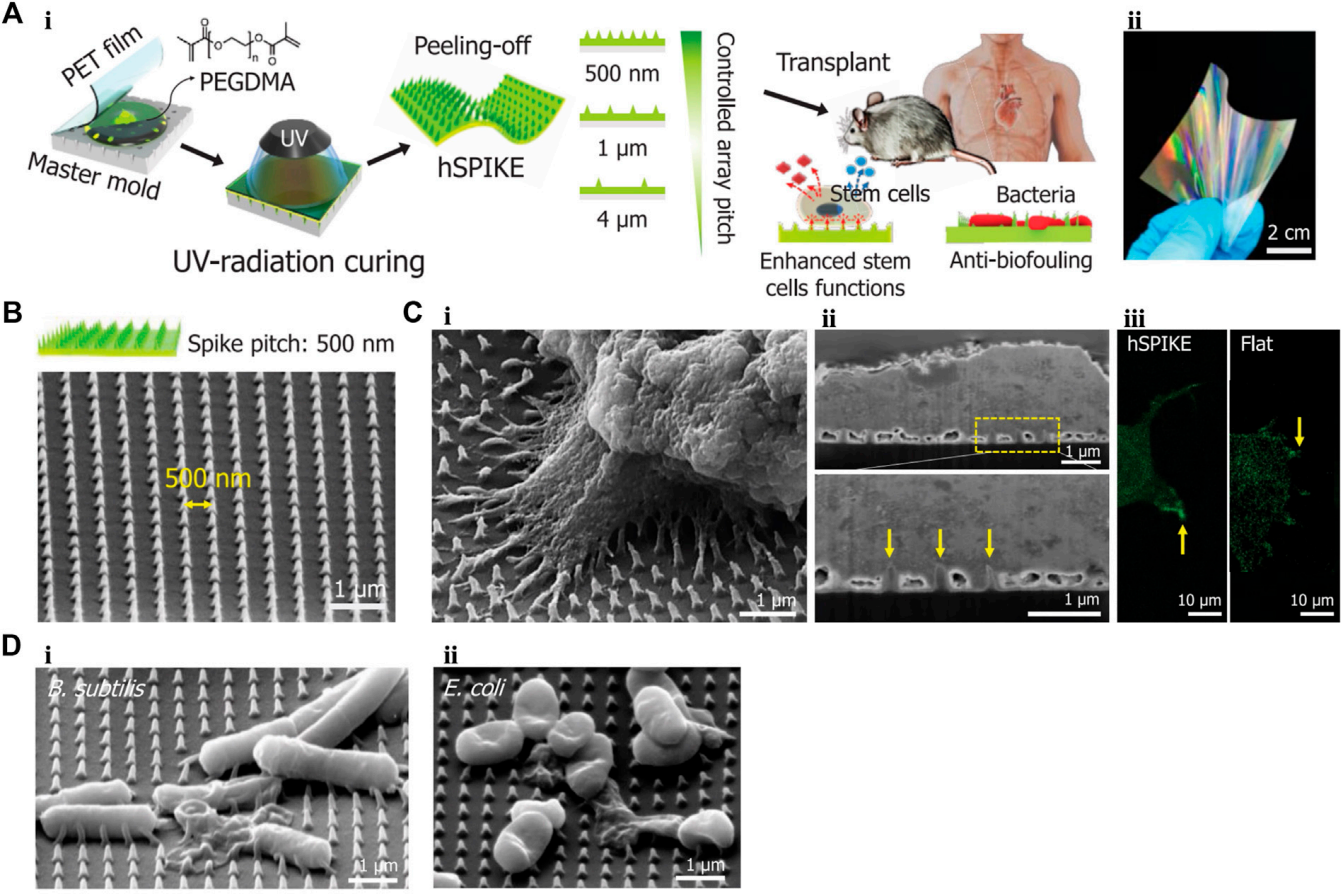
**(A) (i)** Schematic representation of fabrication process of the hSPIKE patch. **(ii)** Photograph of the hSPIKE patch. **(B)** Scanning electron micrograph (SEM) image of the hSPIKE. **(C) (i)** SEM of Dental pulp stem cells (DPSCs) cultured on the hSPIKE, **(ii)** focused ion beam (FIB)-SEM cross-section of DPSCs cultured on the hSPIKE, and **(iii)** fluorescence microscopy images of DPSCs cultured on the hSPIKE (left) and flat substrates (right). **(D)** SEM of **(i)** *B. subtilis* and **(ii)** *E. coli* cultured on the hSPIKE. PET, polyethylene terephthalate; hSPIKE, hydrogel nanospike array; *B. sutilis, Bacillus subtilis*; *E. coli, Escherichia coli*. Reprinted with permission from Park et al. (2019). Copyright: 2019 American Chemical Society.

Based on these advantages of 1D nanomaterials, Park et al. (2019) designed a biocompatible PEG dimethacrylate-based hydrogel patch (Figure 5). This patch possessed a nanospike (hSPIKE) arrays with tapered tips. This transplantable platform promoted the osteogenic, chondrogenic, and adipogenic differentiation of dental pulp stem cells *in vitro* without altering cell viability. Furthermore, the hSPIKE arrays exhibited efficient bactericidal effects against both Gram-positive and -negative bacteria. Compared with a flat patch, the hSPIKE patch significantly promoted the healing of mouse cranial bone defect while preventing bacterial infections.

## 6 Effect on Bone Healing

The impact of any antibiotic-laden-biomaterial (ALB) implanted in a fracture or bone defect on bone regeneration must be addressed. Hydrogels themselves, as foreign materials, and high local concentrations of antimicrobial agents may influence tissue healing (Ter Boo et al., 2018). Studies have investigated bone regeneration following ABL implantation *in vitro* (Ter Boo et al., 2018; Li et al., 2020a; Boot et al., 2020; Tao J. et al., 2020; Vallejo Diaz et al., 2020; Eltawila et al., 2021; Foster et al., 2021; Sun et al., 2021). However, to our knowledge, limited *in vivo* studies have evaluated the influence of hydrogels on bone healing.

Ter Boo et al. (2018) observed fracture healing in groups treated with HA-pNipam hydrogel alone in the absence of bacterial contamination using a rabbit humeral fracture model. Implantation of HA-pNipam hydrogel showed no significant effect on clinical or biological responses, including weight, CRP levels, and WBC count. No significant differences in relative stiffness were observed 4 weeks after hydrogel application. Compared with contralateral non-fractured humerus, the mean relative stiffness values with and without HA-pNipam hydrogel were 49%–67%, respectively. Contact radiographs and histopathological analyses in both groups revealed callus formation at both the *cis* and *trans* sides of the humerus, and at the interface between the screws and intramedullary cavity. Compared to the control group, the HA-pNipam group presented less bone callus formation only at the *cis* side, where the HA-pNipam hydrogel was injected. Another study investigating the effects of a DAC^®^ hydrogel coating on bone apposition at a titanium implant surface *in vivo*, showed that, compared with the group without coating, the hydrogel coating loaded or unloaded with 2% vancomycin neither affected the volume and timing of bone apposition, nor induced an inflammatory response (Boot et al., 2017). The results of these two studies showed that HA-pNipam and DAC^®^ hydrogels did not affect bone regeneration. In future, more research is needed on other hydrogels.

## 7 Hepatorenal Toxicity

Hepatorenal side effects of hydrogels in bone infection have been reported (Changez et al., 2005; Overstreet et al., 2015; Johnson et al., 2018; Johnson et al., 2019; Li et al., 2020b; Foster et al., 2021). In rabbits, Li et al. (2020a) have found that, compared with the blank group, those treated with a VH-HyA-CS/β-GP hydrogel-laden TCP scaffold showed no significant difference in aspartate aminotransferase (AST) and blood urea nitrogen (BUN) levels at 1, 4, and 7 days, whereas alanine aminotransferase and serum creatinine (SCr) levels increased slightly at 4–7 days. In rats, no differences in BUN, SCr, and uric acid (UA) levels were found between the Ti-RP/PCP/RSNO and control groups, while systemic vancomycin treatment increased SCr and UA levels and induced renal toxicity (Li et al., 2020b). Additionally, liver enzyme tests and histological analyses of the liver and kidneys supported the safety of lysostaphin-delivering PEG hydrogels designed by Johnson et al. (2018). These results support the safety of the application of these local antimicrobial agent delivery hydrogel systems.

## 8 Outlooks

Collectively, available hydrogel designs have demonstrated the superior ability to prevent or treat OM, compared to the clinical gold standard of ALBC. However, there remains several challenges in the clinical applications. First, potential resistance to present agents remains a concern. Co-delivery of different agents may represent a suitable approach to overcome this challenge. Alternative strategies, such as mAb treatment and conjugation of bisphosphonates to conventional antibiotics, are increasingly attractive for the treatment of OM (Sedghizadeh et al., 2017; Masters et al., 2019). Hydrogel loading with these agents would increase the number of therapeutic strategies. Second, whether the hydrogel can be applied once in a one-stage exchange procedure remains to be determined. Eliminating the need for a second revision surgery would be an attractive prospect (Boot et al., 2021). Thus, future work should focus on engineering smart or intelligent hydrogel delivery systems that are responsive to multiple stimuli, and outstanding breakthroughs in 4D printing (Chu et al., 2020). Third, present studies have focused on the OM induced by *S. aureus*. Different hydrogel antimicrobial approaches should be investigated for infections by a diverse range of species. Fourth, to date, only a few drug-laden hydrogels have been used successfully in the clinic. More studies are needed to develop an optimal hydrogel system for OM.

## 9 Conclusion

OM remains a major challenge due to its complicated microenvironment. An in-depth understanding the mechanism of OM will help to develop better treatment strategies. Loading antimicrobial agents into hydrogels, even co-incorporating osteo-inductive materials and other adjuvants, is a promise strategy. This review discussed current hydrogel designs and their applications to improve the biological response to OM *in vivo*, thereby providing insights into the development of novel prevention and treatment options for OM.
