# Supplementary material for: Recent Advances in Multifunctional Hydrogels for the Treatment of Osteomyelitis
Source: Front Bioeng Biotechnol. 2022 Apr 25;10:865250. doi: 10.3389/fbioe.2022.865250 (PMC9081433; doi:10.3389/fbioe.2022.865250)
Supplement: Supplementary file 1 [file Table1.docx]

# Supplementary Table 1. Summary of Hydrogels Used for in *In Vivo* OM Studies on OM

| Hydrogel | Anti-microbial agents | Release pattern | Antimicrobial agents release time  (*In vitro*) | Animal | Location | Model | Species | *In vivo* application | Findings  (*in vivo*) | References |
| --- | --- | --- | --- | --- | --- | --- | --- | --- | --- | --- |
| HA-pNipam | GNT | / | / | Rabbit | Humerus | Open fracture ODRI | *S. aureus* | Hydrogel implant | Local prophylaxis with the hydrogel achieved a 0% culture positive rate without requiring postoperative systemic prophylaxis. The rate was 16.6% in the traditional prophylaxis group. | (Vallejo Diaz et al., 2020) |
| CS β-glycerophosphate HyA | VCM | Sustained or burst and sustained (depended on the concentration of hyaluronidase) | >25 days | Rabbit | Femoral condyles | OM defect | *S. aureus* | Hydrogel-loaded scaffold implant | Compared to 1-week intramuscular injection of antibiotic, the intelligent drug-release system was highly effective at inhibiting bone destruction and inflammation at 30 days. | (Li et al., 2020a) |
| GEL | GNT | Burst and sustained | 28 days | Rat | Femoral condyles | OM defect | *S. aureus* | Hydrogel-loaded scaffold implant | OM was cured at 3 weeks. | (Wu et al., 2013) |
| PEG-4MAL | Lysostaphin | Sustained | ~ 80% of lysostaphin was released w/wo collagenase at 4 and 2h respectively， almost 80% and 60% BMP-2 was released w/wo collagenase at 4h | Mouse | Radius | OM defect | *S. aureus* | Hydrogel implant | BMP-2–loaded lysostaphin-delivering hydrogels eliminated infection and promoted defect repair. | (Johnson et al., 2019) |
| PEG-4MAL | Bacteriophage | / | / | Mouse | Radius | OM defect | *P. aeruginosa* | Hydrogel implant | Hydrogels reduced pathogen counts by 4.7-fold at the infection site compared to bacteriophage-free hydrogels at 7 days | (Wroe et al., 2020) |
| Poly (acrylic acid) GEL | GNT /VCM | Burst and sustained | / | Rabbit | Femoral condyles | OM defect | *S. aureus* | Hydrogel implant | Infections were cured at 6 weeks. | (Changez et al., 2005) |
| Polypropylene glycol NF polysorbate-20 NF (Tween 20) methyl paraben NF xanthan gum (Kelco K9B310) Ultrez 10 (carbopol NF) glycerin | Bismuth thiols | / | / | Rat | Femur | OM defect | *S. aureus* | Hydrogel implant | With systemically administered cefazolin, bismuth thiols loaded hydrogel decreased infection. | (Penn-Barwell et al., 2015) |
| Collagen | GNT | Sustained | / | Rabbit | Mandibula | OM | *S. aureus* | Hydrogel implant | Combining debridement with a single injection of antibiotic-loaded hydrogel suppressed experimental OM compared to the untreated control and multiple systemic doses of IM GNT solution. | (Eltawila et al., 2021) |
| ALG | Bacteriophage | / | / | Rat | Femur | OM | *S. aureus* | Hydrogel implant | Bacteriophages delivered via ALG hydrogel successfully reduced soft tissue infection, but not bone infection at 1-day post-implantation. | (Cobb et al., 2019) |
| ALG HyA | VCM | VCM sustained BMP burst and sustained | >6 weeks | Rat | Femur | OM | *S. aureus* | Hydrogel implant | VCM/BMP2-loaded-ALG/HyA hydrogel effectively suppressed *S. aureus* and enhanced bone regeneration. | (Aldrich et al., 2019) |
| CS | VCM NPs | Sustained | >25 days | Rabbit | Tibia | OM | *S. aureus* | Hydrogel implant | The VCM-NPs/Gel demonstrated excellent anti-infective properties and accelerating bone repair under OM conditions. | (Tao et al., 2020b) |
| mPEG-PLGA | Teicoplanin | Sustained | >28 days | Rabbit | Femur | OM | *S. aureus* | Hydrogel implant | Sustained release of teicoplanin from mPEG-PLGA hydrogel efficiently treated OM at 8 weeks. | (Peng et al., 2010) |
| PEGDMA | / | / | / | Mouse | Cranium | OM | *P. aeruginosa* | Hydrogel patch | The hSPIKE patch presented efficient bactericidal activity compared to a planar patch. | (Park et al., 2019) |
| RADA16  peptide | Tet213 | Burst and sustained | 28 days | Rabbit | Tibia | OM | *S. aureus* | Hydrogel implant | RADA16-AMP self-assembling peptide inhibited the pathogen proliferation and promoted bone formation. | (Yang et al., 2018a) |
| CS | AgNPs | Burst and sustained | 7 days | Rabbit | Tibia | ODRI | *S. aureus* | Coating | The Ag-laden hydrogel did not present any antibacterial effect as compared to the hydrogel alone at 28 days. | (Croes et al., 2018) |
| DAC^®^ | VCM | Burst | / | Rabbit | Femur | ODRI | MRSA | Coating | Local bacterial load reduced, ranging from 72% to 99 %. | (Giavaresi et al., 2014) |
| DAC^®^ | VCM | / | / | Rat | Femur | ODRI | MRSE | Coating | Local VCM hydrogel group presented scarce dispersed cocci within areas of new bone formation compared with systemic treatment. | (Lovati et al., 2016) |
| DAC^®^ | VCM | / | / | Rabbit | Tibia | ODRI | *S. aureus* | Coating | The VCM-laden DAC^®^ group presented a lower infection rate and higher average percentage of bone implant contact at 28 days. | (Boot et al., 2020) |
| GEL ALG | VCM/GNT | Sustained | >120h | Rat | Femur | ODRI | MRSA | Coating | VCM-impregnated TGase cross-linked GEL/ALG hydrogel was effective at alleviating ODRI and bone regeneration. Release of antibiotics slowed with higher TGase concentrations. | (Sun et al., 2021) |
| HA-pNipam | VCM/GNT | Burst and sustained | >336 h | Sheep | Tibia | ODRI | MRSA | Coating | Local antibiotic-laden hydrogel ramatically increased treatment success rates beyond current clinical practice (ALBC). | (Boot et al., 2021) |
| HA-pNipam | VCM/GNT | Burst and sustained | / | Sheep | Tibia | ODRI | MRSA | Coating | For single-stage revisions, antibiotic-loaded hydrogels had the same effect as ABLC, with more practical benefits than ABLC. | (Foster et al., 2021) |
| HA-pNipam | GNT | Burst | < 7 days | Rabbit | Humerus | ODRI | *S. aureus* | Hydrogel implant | The bacterial burden was cleared in all inoculated rabbits. | (Ter Boo et al., 2016) |
| HA-pNipam | / | / | >5 days | Rabbit | Humerus | ODRI | *S. aureus* | Hydrogel implant | Culture negativity was achieved at 4 weeks | (Ter Boo et al., 2018) |
| PEG | VCM | Sustained | >3 weeks | Rabbit | Tibia | ODRI | *S. aureus* | Coating | The antibiotic hydrogel coating exhibited a good antimicrobial capability. | (Li et al., 2017) |
| PEG-4MAL | Lysostaphin | Sustained | 24h | Mouse | Femur | ODRI | *S. aureus* | Hydrogel implant | Lysostaphin delivering hydrogel group showed the equivalent bone formation and mechanical properties to those of uninfected fracture group, whereas non hydrogel carrier group did not. | (Johnson et al., 2018) |
| PNDJ | GNT | Burst and sustained | >7 days | Rabbit | Radius | ODRI | *S. aureus* | Hydrogel implant | No infection was found in the GNT-load hydrogel group 4 weeks after debridement. | (Overstreet et al., 2015) |
| PVA CS | RSNO | Adjustable | / | Rat | Femur | ODRI | MRSA | Coating | Via NIR, the photothermal effect disrupted bacterial integrity and eliminated the MRSA burden in a synergistic manner with the simultaneous generation of ·ONOO− and ·O2-. | (Li et al., 2020b) |
| ME-HyA ME-GEL | MΦs +rifampin +daptomycin | Burst and sustained | >14 days | Mouse | Cranium | Bone defect | *S. aureus* | 3D bioprinting hydrogel-loaded scaffold implant | Bacterial burden associated with craniotomy was reduced. | (Aldrich et al., 2019) |
| PEG | AgNPs | / | / | Rat | Maxillary | Bone defect | / | Hydrogel implant | Defects were cured. | (Xu et al., 2018) |
| PLA-DX-PEG | Teicoplanin | Burst and sustained | >21days | Rat | Cranium | Bone defect | / | Hydrogel implant | The ability of rhBMP2 to repair cranial defects was not impact by the co- encapsulated antibiotics. | (Suzuki et al., 2006) |
| PNDJ | Tobramycin + VCM | Burst and sustained | / | Rabbit | Femur knee  biceps femoris | *In-vivo* pharmacokinetic studies | / | Hydrogel implant | The drug levels were depending on the site of application but not the polymer formulations. The local drug concentrations were maintained over estimated biofilm-eradicating levels for more than 24 hours. | (Overstreet et al., 2019) |
| PLGA-PEG-PLGA | DINH liposome | Burst and sustained | >120 h | Rabbit | / | Intra-articular pharmacokinetic studies | / | Hydrogel implant | Compared with liposome-free hydrogel, liposome-in-hydrogel hydrogel exhibited a sustained release profile. | (Liu et al., 2019) |
| DAC^®^ | VCM | / | / | Rabbit | Tibia | Evaluation of the effect of implant coating on osseointegration and hematologic parameters. | / | Coating | Loaded or unloaded with 2% (w/v) vancomycin, the hydrogel coating on titanium rods had no effect on the volume or timing of bone apposition. | (Boot et al., 2017) |
| DAC^®^ | GNT /VCM  /amikacin /tobramycin /NAC /sodium salicylate | Burst | < 96 h | Human /rabbit | Femur /tibia | Press-fit insertion test | / | Coating | After press-fit insertion, 80% of hydrogel were retained on the prothesis. | (Drago et al., 2014) |

CS, chitosan; HyA, hyaluronic acid; GEL, gelatin; PEG, poly(ethylene glycol); PEG-4MAL, four-arm poly(ethylene glycol)-maleimide; mPEG, poly(ethylene glycol) monomethyl ether; ALG, alginate; PEGDMA, poly(ethylene glycol) dimethacrylate (PEGDMA); PLGA, poly(lactic-co-glycolic acid); AMP, antimicrobial peptide; PNDJ, poly(N-isopropylacrylamide-co-dimethyl-γ-butyrolactone acrylate-co-Jeffamine® M-1000 acrylamide); PVA, modificatory polyvinyl alcohol; RSNO, NO donor of S-nitrosuccinic acid; ME-HyA, methacrylated hyaluronic acid; ME-GEL, methacrylated gelatin; PLA, poly-D,L-lactic acid; DAC, Disposable Antibacterial Coating; DINH, N′-Dodecanoylisonicotinohydrazide; MRSA, methicillin-resistant *S. aureus*; GNT, gentamicin; VCM, vancomycin; MΦs, macrophages; BMP, bone morphogenetic protein; OM, osteomyelitis; ODRI, orthopedic device-related infections; MIC, minimum inhibitory concentration; MBC, minimum bactericidal concentration; NPs, nanoparticles; /, not mentioned.
